# IgG deposits in the mesangium and capillary loops predict poor renal outcome in patients with IgA nephropathy: a single-center retrospective study

**DOI:** 10.1080/0886022X.2020.1811120

**Published:** 2020-09-01

**Authors:** Siqi Peng, Wen Lu, Xiao Jiang, Xingxin Xu, Yonggui Wu

**Affiliations:** Department of Nephrology, the First Affiliated Hospital of Anhui Medical University, Hefei, China

**Keywords:** IgA nephropathy, renal pathology, IgG co-deposition, IgG deposit location

## Abstract

**Background:**

Glomerular IgG deposition in patients with IgA nephropathy (IgAN) has been shown to be associated with poor renal survival; however, most published studies to date are too small-scale and inconsistent to provide guidance for clinical practice.

**Methods:**

Based on renal biopsy findings, 742 patients were divided into the following groups: (i) IgA deposition alone (IgA) vs IgA + IgG deposition (IgA + IgG) and (ii) IgG co-deposition confined to the mesangium vs mesangium + capillary loops (CLs). The clinicopathological variables at biopsy and renal outcome were assessed.

**Results:**

Of the 742 patients, 182 had IgG co-deposition and 51 had IgG deposits in the mesangium + CLs. Patients with IgG co-deposition were associated with severe clinical and pathological lesions, especially those with a location of IgG deposits in the mesangium +CLs. Kaplan–Meier analysis revealed that a lower renal cumulative survival rate was present in both patients with IgG co-deposition and those with a location of IgG deposits in the mesangium + CLs (all *p* < 0.05). Moreover, patients with a higher intensity of glomerular IgG deposits or C3 deposits or C1q deposits were also associated with a lower survival rate. A multivariate Cox regression model identified the location of IgG deposits in the mesangium + CLs as an independent risk factor for poor prognosis (*HR*, 2.11; 95% CI: 1.06–4.18; *p* = 0.005).

**Conclusions:**

Glomerular IgG co-deposition and the location of glomerular IgG deposits in the mesangium + CLs were both associated with adverse renal outcomes, but only the location of glomerular IgG deposits in the CLs was an independent risk factor for poor prognosis in IgAN.

## Introduction

IgA nephropathy (IgAN), the most common form of primary glomerulonephritis worldwide, is characterized by the dominant or co-dominant presence of immunoglobulin A (IgA) by immunofluorescence microscopy in the glomerular mesangium [1]. However, the exact mechanism of IgAN remains obscure, it is thought to be an immune-related disease with overproduction of galactose-deficient IgA1 which was influenced by genetic variation at the C1GALT1, ST6GALNAC2 and C1GALT1C1 gene [2–4]. A recent study showed that the decreased HECW1 expression is linked with the overproduction of Gd-IgA1, thereby providing a new regulatory mechanism of IgAN that can explain the aberrant glycosylation of IgA1 responsible for the pathogenesis of the disease [5].

Though many cases of IgAN patients carry a good prognosis, many people progress to end-stage renal disease (ESRD) slowly. A greater understanding of the risk factors associated with IgAN is therefore required to guide treatment decisions. It has been well documented that a number of clinicopathological features are associated with poor prognoses, such as old age, hypertension, massive proteinuria, reduced estimated glomerular filtration rate at biopsy, and Oxford-MESTC lesions [6–10]. Some studies have also found that lifestyle factors, such as drinking alcohol, decreased physical activity, are associated with a high risk of progression to end-stage renal disease [11]. However, few studies have examined the effect of immunofluorescence findings of patients with IgAN on the renal outcome. In most cases with IgAN, immunofluorescence staining suggested that cases presenting with IgA deposition alone comprise <25%, and co-deposits of IgG, IgM, and complement C3 are also observed [12].

In an early animal model, the mesangial co-deposition of IgA together with IgG aggravated glomerular inflammation in a complement-dependent fashion [13]. A Japanese study suggested that IgAN with glomerular IgG co-deposition presented more severe clinical features and a low complete remission rate; however, these alterations were independent of the location of glomerular IgG deposits [14]. Another recent study indicated that IgG co-deposition and the location of glomerular deposits in the peripheral capillary walls were both associated with severe clinicopathological lesions on renal biopsy, but only the location of glomerular deposits in the peripheral capillary walls was closely related to worse renal outcomes [15]. However, the majority of these studies have been too small-scale to produce adequate evidence to inform therapeutic decision-making.

Therefore, we initiated this large-scale, single-center study to assess whether the presence of IgG co-deposition or the location of IgG deposits was clinically significant in patients with IgAN. Meanwhile, we incorporated glomerular IgG deposits and the location of IgG deposits into the Oxford-MESTC classification and evaluated their prognostic value.

## Method and materials

### Study subjects

**Figure 1.**
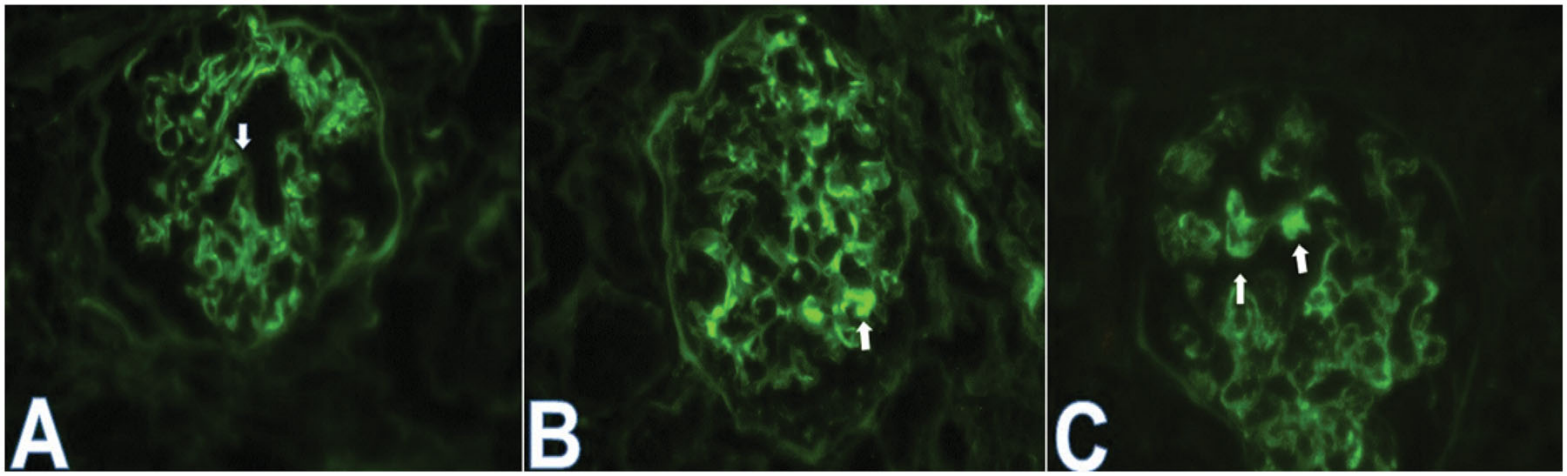
(A) Mesangium staining for IgG (1+) (×400). (B) Mesangium staining for IgG (2+) (×400). (C) PCW (left arrow) and mesangium (right arrow) IF staining for IgG(2+) (×400).

This study was a hospital-based retrospective analysis of the clinicopathological data of IgA nephropathy (IgAN). Between January 2015 and September 2019, 921 patients were diagnosed with IgAN. IgAN was defined as glomerulonephritis with IgA as the sole or main glomerular immunofluorescence finding. The exclusion criteria were as follows: (i) patients aged <18 years or >75 years or with a follow-up shorter than 6 months; (ii) patients with secondary IgA deposition diseases, such as systemic lupus erythematosus (SLE), ANCA-associated vasculitis, allergic purpura, ankylosing spondylitis renal damage and psoriatic renal damage; (iii) patients with serious underlying diseases and comorbidities, such as diabetes, chronic heart failure, and hypertension, chronic liver diseases; (iv) patients with whose biopsy showed less than 10 glomeruli in a light microscopy analysis. Overall, a total of 742 patients were included in this study. These 742 patients were divided into groups showing IgA without IgG co-deposition (*n* = 560) and IgA + IgG co-deposition (*n* = 182) by IF evaluation. Patients with IgAN with glomerular IgG co-deposition were also categorized into two groups according to the location of IgG deposits: IgG deposits confined to the mesangium (*n* = 131) and IgG deposits in the mesangium + CLs (*n* = 51) by IF evaluation (Figure 1).

### Data collection

All clinicopathological data were obtained at the time of renal biopsy, including age, sex, clinical course, gross hematuria, blood pressure, and medications. We collected laboratory data including hemoglobin, serum IgA/C3, serum albumin/globulin, serum creatinine, estimated glomerular filtration rate (eGFR), uric acid, triglyceride, total cholesterol and 24-h urinary protein at the time of renal biopsy.

All pathological data were extracted from the pathology reports. Two pathologists evaluated the renal biopsy specimens independently. Routine histologic assessments were based on hematoxylin-eosin, periodic acid-Schiff, Masson’s trichrome, methenamine silver, and Congo red for light microscopy. Direct immunofluorescence for IgA, IgG, IgM, C3, and C1q was performed on frozen tissue sections, with results presented semiquantitatively from 0 to 4+. The histologic severity of glomerular lesions was graded using H.S. Lee^,^s glomerular system [16]. Meanwhile, histopathological classification was also made according to the MEST-C score of the Oxford criteria [6]. Pathological features also included global glomerulosclerosis, renal vascular lesions (arteriole wall thickening and small vessel hyalinosis), and interstitial inflammation. Interstitial inflammatory lesions were evaluated semiquantitatively on the basis of the affected cortical area: none (score 0), mild (score 1; <25%), moderate (score 2; 25–49%), and severe (score 3; ≥50%) [17].

### Definitions

Mean arterial pressure (MAP) was defined as diastolic pressure plus one-third of the pulse pressure. Hypertension was defined as systolic blood pressure >140 mmHg and/or diastolic blood pressure >90 mmHg [18]. Anemia was defined as hemoglobin <110 g/L for females and 120 g/L for males [19]. The estimated glomerular filtration rate (eGFR) was calculated using the four-variable Modification of Diet in Renal Disease Study (MDRD) formula in adults [20]. The primary combined outcome was the doubling of serum creatinine from baseline values, 50% loss of GFR from baseline values, or the occurrence of end-stage renal disease (including eGFR < 15 mL/min/1.73 m^2^ or receiving long-term dialysis or kidney transplantation or death) during the follow-up period.

### Statistical analysis

Statistical analyses were performed using SPSS software (version 23.0, IBM SPSS). Continuous variables with normal distribution were presented as the means ± SD and compared using the *t*-test. Non-normally distributed continuous variables were expressed in medians with interquartile ranges and compared using the Mann–Whitney U test. Categorical variables are summarized as numbers and percentages and compared using the chi-squared test or Fisher’s exact test or the Mann–Whitney U test. Cumulative kidney survival curves were derived using the Kaplan–Meier method, and the differences between curves were analyzed using a log-rank test. Multivariate analysis by using the Cox proportional hazards regression model was performed to identify the independent prognostic factors. All *p*-values were two-sided, and *p-*values less than 0.05 were considered to indicate statistical significance.

## Results

### Patients

Between January 2015 and September 2019, 742 eligible patients were recruited. Of the 742 patients, 182 (24.5%) he mesangium and IgG deposits within CLs, respectively. The median age was 36 (IQR 18–68) years, and the patients were mainly young adults. Among those where specific sex was indicated, 56.2% were female, and the male-to-female sex ratio was 1:1.28. The median follow-up time was 31 months (range 6–61), during which 75.9% received inhibitors of the renin-angiotensin system (RASI), 28.2% received corticosteroids and 2.4% received immunosuppressants. A total of 324 (43.7%) patients had hypertension at biopsy, and 95 (12.8%) had anemia at biopsy. Gross hematuria was noted in 14.6% of patients.

### Clinicopathological findings at renal biopsy

The clinicopathological features of 742 patients enrolled in the present study are summarized in Tables 1 and 2. Patients with IgAN with IgG co-deposition showed a higher level of total cholesterol and a higher proportion of E1 and more positive instances of IgM and C1q staining than patients without IgG co-deposition (all *p* < 0.05). There was no other baseline difference between the IgA and IgA + IgG groups (all *p* > 0.05). The proportions of patients receiving renin-angiotensin system inhibitors, corticosteroids, and immunosuppressive treatments were similar in both groups.

**Table 1.** Baseline clinical characteristics and treatment, IgAN by IgG codeposition, IgAN by glomerular IgG deposit location.

Clinical characteristics	IgG codeposition		*p*-Value	Glomerular IgG deposit locaion		*p*-Value
	IgA-IgG (*n* = 182)	IgA (*n* = 560)		Mesangium only (*n* = 131)	Mesangium + CLs (*n* = 51)	
Female/male	77/105	248/312	0.640	54/77	23/28	0.634
Age (years)	37 (28, 47)	35 (28, 45)	0.569	37 (28, 47)	37 (29, 46)	0.857
Course (months)	5.0 (1.0, 12.0)	6.0 (1.0, 24.0)	0.102	6.0 (1.0, 12.0)	4.0 (0.5, 9.0)	0.024
Gross hematuria (%)	24 (13.2%)	84 (15.0%)	0.547	15 (11.5%)	9 (17.6%)	0.267
MAP (mmHg)	102.2 ± 14.3	101.4 ± 13.9	0.489	99.8 ± 13.5	108.2 ± 14.8	0.000
Hypertension (%)	80 (44.0%)	244 (43.6%)	0.987	47 (35.9%)	33 (64.7%)	0.000
Hemoglobin (g/L)	129.9 ± 18.3	130.5 ± 17.9	0.720	128.5 ± 17.6	133.7 ± 19.8	0.080
Anemia (%)	24 (13.2%)	71 (12.7%)	0.859	18 (13.7%)	6 (11.8%)	0.723
Serum IgA/C3	2.67 ± 1.22	2.66 ± 1.00	0.916	2.73 ± 1.15	2.54 ± 1.40	0.345
Serum A/G	1.43 ± 0.30	1.46 ± 0.28	0.245	1.46 ± 1.29	1.36 ± 0.328	0.045
Proteinuria (g/day)	1.1 (0.5, 2.0)	1.0 (0.6, 1.8)	0.477	0.9 (0.5, 1.9)	1.3 (0.6, 3.6)	0.033
SCr (μmol/L)	97.1 ± 44.1	92.8 ± 49.4	0.305	90.9 ± 37.8	112.8 ± 54.5	0.010
eGFR (mL/min/1.73 m^2^)	85.55 ± 32.03	90.20 ± 29.52	0.071	89.80 ± 30.05	74.63 ± 34.62	0.004
eGFR^a^	91 (50.0%)	257 (45.9%)	0.335	57 (43.5%)	34 (66.7%)	0.005
UA (μmol/L)	376.8 ± 109.1	372.4 ± 100.9	0.621	370.9 ± 111.3	391.8 ± 102.9	0.249
TG (mmol/L)	1.5 (1.2, 2.4)	1.6 (1.2, 2.3)	0.776	1.6 (1.1, 2.3)	1.8 (1.2, 2.7)	0.166
TC (mmol/L)	5.0 (4.1, 5.8)	4.6 (3.9, 5.4)	0.001	4.8 (4.1, 5.5)	5.5 (4.5, 6.4)	0.005
Treatment (%)						
RASI	145 (79.7%)	418 (74.6%)	0.168	106 (82.9%)	39 (76.5%)	0.503
Corticosteroid	52 (28.6%)	157 (28.0%)	0.889	31 (23.7%)	21 (41.2%)	0.019
Immunosuppressants	5 (2.7%)	13 (2.3%)	0.782	3 (2.3%)	2 (3.9%)	0.620

Values for continuous variables are given as median (interquartile range) or mean ± standard deviation; values for a categorical variable, as number (percentage) or absolute number.

CLs: capillary loops; MAP: mean arterial pressure; Serum A/G: serum albumin/globulin; SCr: serum creatinine; eGFR: estimated glomerular filtration rate; eGFR^a^: eGFR < 90 mL/min/1.73 m^2^; UA: uric acid; TG: triglycerides ; TC: total cholesterol; RASI: renin-angiotension system inhibitor.

**Table 2.** Baseline histological characteristics and renal outcome, IgAN by IgG codeposition, IgAN by glomerular IgG deposit location.

Histological characteristics	IgG codeposition		*p*-Value	Location of IgG deposition		*p*-Value
	IgA-IgG (*n* = 182)	IgA (*n* = 560)		Mesangium only (*n* = 131)	Mesangium + CLs (*n* = 51)	
IgM (%)	101 (55.5%)	259 (46.3%)	0.030	66 (50.3%)	35 (68.6%)	0.026
C3 (%)	173 (95.1%)	535 (95.5%)	0.788	126 (96.2%)	476 (92.2%)	0.487
C1q (%)	30 (16.5%)	29 (5.1%)	0.001	16 (12.2%)	14 (27.4%)	0.024
Oxford classification						
M1 (%)	182 (100%)	560 (100%)	1.000	131 (100%)	51 (100%)	1.000
E1 (%)	56 (30.8%)	140 (25.0%)	0.000	32 (24.4%)	24 (47.1%)	0.003
S1 (%)	95 (52.2%)	300 (53.6%)	0.747	71 (54.2%)	24 (47.1%)	0.386
T1 (%)	93 (51.1%)	283 (50.5%)	0.804	60 (45.8%)	33 (64.7%)	0.025
T2 (%)	13 (7.1%)	46 (8.2%)		9 (6.9%)	4 (7.8%)	
C1-2 (%)	74 (40.7%)	224 (40.0%）	0.875	49 (37.4%)	25 (49.0%)	0.152
Interstital inflammation (%)						
Mild	77 (42.3%)	256 (45.7%)	0.422	62 (47.3%)	15 (29.4%)	0.028
Moderate	66 (36.3%)	212 (37.9%)	0.700	47 (35.9%)	19 (37.3%)	0.862
Severe	39 (21.4%)	92 (16.4%)	0.124	22 (16.8%)	17 (33.3%)	0.015
Arteriole wall thickening (%)	146 (80.2%)	468 (83.6%)	0.121	104 (79.4%)	42 (82.4%)	0.825
Small vessel hyalinosis (%)	4 (2.2%)	11 (2.0%)	0.769	1 (0.8%）	3 (5.9%)	0.000
Glomeruli with GS (%)	54 (29.7%)	165 (29.5%）	0.958	38 (29.0%）	16 (31.4%)	0.117
Combined outcome (%)			0.000			0.000
ESRD	12 (6.6%)	7 (1.2%)		5 (3.8%)	7 (13.7%)	
Double SCr	17 (9.3%)	31 (5.5%)		7 (5.3%)	10 (19.6%)	
50% loss in eGFR	6 (3.3%)	4 (10.7%)		2 (1.5%)	4 (7.8%)	

Values are presented as number (percentage).

CLs: capillary loops; M0: mesangial hypercellularity score of ≤0.5; M1: mesangial hypercellularity score of >0.5; E0: absence of endocapillary hypercellularity; E1: presence of endocapillary hypercellularity ; S0: absence of segmental glomerulosclerosis; S1: presence of segmental glomerulosclerosis; T0: tubular atrophy/interstitial fibrosis ≤25% of cortical area; T1: tubular atrophy/interstitial fibrosis 26–50% of the cortical area; T2: tubular atrophy/interstitial fibrosis >50% of the cortical area; C0: absence of crescents; C1-2: presence of crescents. GS: global sclerosis; ESRD: end-stage renal disease, including eGFR <15 mL/min/1.73 m^2^ or receiving long-term dialysis or kidney transplantation; double SCr: doubling of serum creatinine concentration.

**Figure 2.**
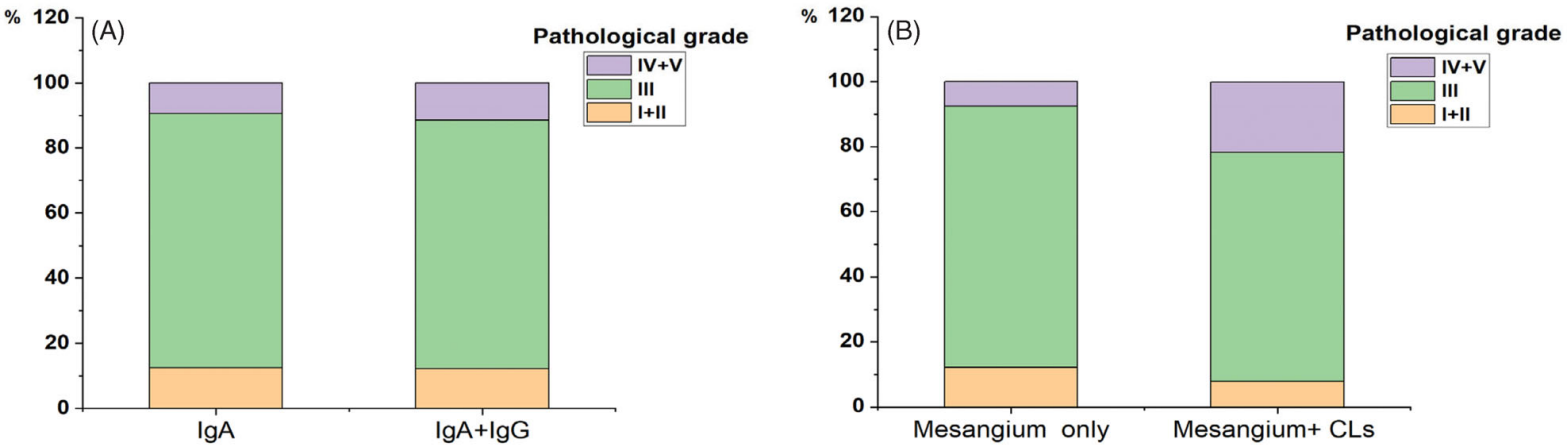
The distribution of renal pathology grade (Lee’s grade) in IgAN. (A) IgA and IgA + IgG (*p* = 0.571). (B) IgG deposits in the mesangium only and IgG deposits in the mesangium + capillary loops (CLs) (*p* = 0.020).

With regard to the location of glomerular IgG deposits, patients with glomerular IgG deposits in the mesagium + CLs were associated with higher levels of MAP, proteinuria, serum creatinine, and total cholesterol and were more likely to have positive instances of IgM and C1q staining, endocapillary hypercellularity (E1), tubular atrophy/interstitial fibrosis (T1-2), small vessel hyalinosis and severe interstitial inflammation infiltration than those with IgG deposits restricted to the mesangium (all *p* < 0.05). Additionally, patients with IgG deposits in the mesangium + CLs had a significant decrease in the eGFR and could be far more prone to receiving corticosteroid treatment compared with those with IgG deposits in the mesangium only (all *p* < 0.05). In terms of histological grade (H.S. Lee’s grade), grade IV and grade V changes were seen more frequently in patients with IgG deposits in the mesangium + CLs than those with IgG deposits restricted to the mesangium (21.6 vs 7.6%, *p* = 0.020; Figure 2). Furthermore, the frequency of glomerular crescents in the kidney biopsy did not differ between patients with IgG deposits in the mesangium + CLs and those with IgG deposits in only the mesangium (49.0 vs 37.4%, *p* = 0.152).

### Clinical outcomes and prognostic value of IgAN with IgG co-deposition and the location of IgG deposits

**Figure 3.**
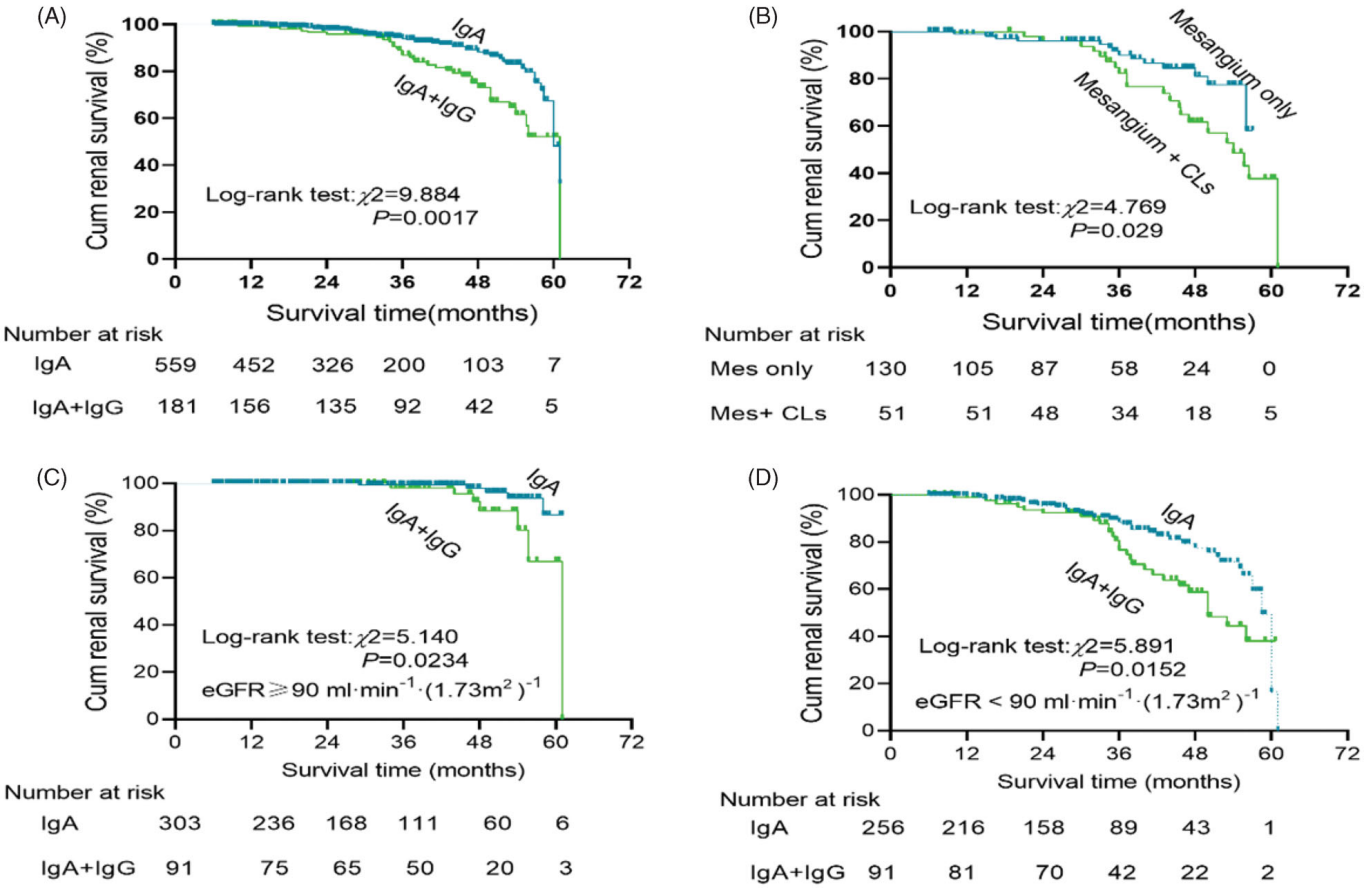
Kaplan–Meier renal survival to compare: (A) IgA (*n* = 560) vs IgA + IgG (*n* = 182); (B) IgG deposits in mesangium Only (*n* = 131) vs IgG deposits in mesangium + CLs (*n* = 51); (C) All patients with eGFR ≥90 mL/min/1.73 m^2^ (*n* = 394):IgA (*n* = 303) vs IgA + IgG (*n* = 91); (D) All patients with eGFR <90 mL/min/1.73 m^2^ (*n* = 348):IgA (*n* = 257) vs IgA + IgG (*n* = 91).

**Figure 4.**
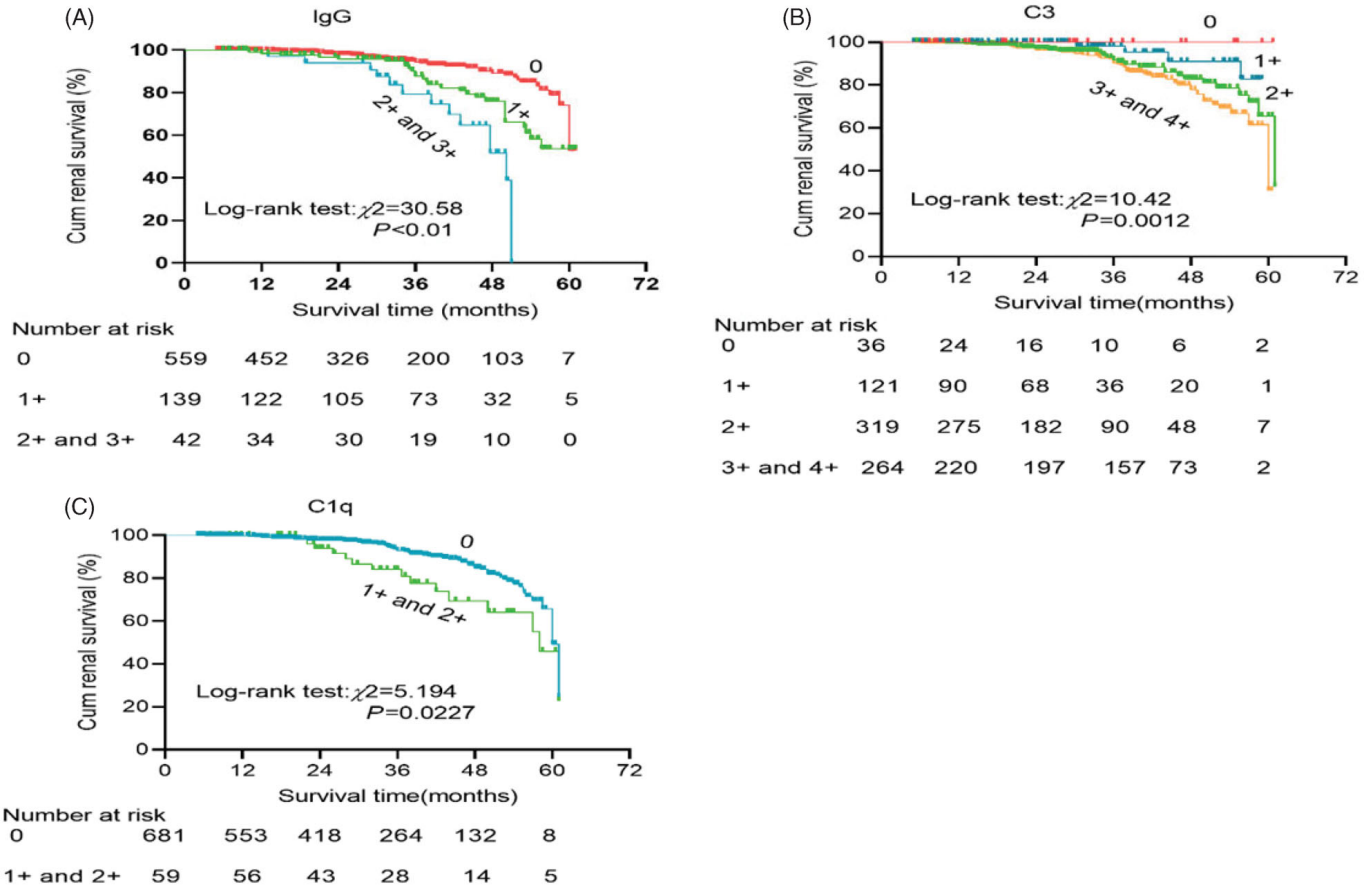
Kaplan–Meier renal survival to compare: (A) The intensity of IgG codeposition: 0 (*n* = 560) vs 1+ (*n* = 140) vs 2+ plus 3+ (*n* = 42). (B) The intensity of C3 deposition: 0 (*n* = 34) vs 1+ (*n* = 122) vs 2+ (*n* = 321) vs 3+ plus 4+ (*n* = 265). (C) The intensity of C1q: 0 (*n* = 683) vs 1+ plus 2+ (*n* = 59).

At the end of follow-up, 25 (3.4%) patients were lost to follow-up, and 77 (10.3%) patients reached the primary combined outcome. Among these patients, the endpoint of 50% loss of eGFR from baseline values was reached in 10 (1.3%), doubling of serum creatinine concentration was reached in 48 (6.4%), and end-stage renal disease was present in 19 (2.5%). Of the 77 patients who had reached primary combined outcomes, 41 did not have IgG co-deposition, 36 did have IgG co-deposition (*p* < 0.01; Table 2). The chance of experiencing the primary combined outcome was 2.02-fold higher in patients with IgG co-deposition compared with those without IgG co-deposition (95% CI: 1.28–3.17, *p* = 0.002; Table 3). Kaplan–Meier survival curves illustrated that the overall survival rate was significantly lower in patients with IgAN with IgG co-deposition than in those without IgG co-deposition (log-rank test: *χ*^2^ = 5.140, *p* = 0.0234; Figure 3(A)). When stratified by renal function, there were significant congruency effects within both groups (all *p* < 0.05; Figure 3(C,D)). Moreover, patients with a higher intensity of glomerular IgG deposits or C3 deposits or C1q deposits were also associated with a lower survival rate (all *p* < 0.05; Figure 4(A–C)).

**Table 3.** Incidence of primary combined outcome, IgAN by IgG codeposition, IgAN by glomerular IgG deposit location.

Biopsy group	Primary renal outcome	Odds ratio (95% CI)	*p*-Value
IgA + IgG vs IgA	41 (5.5%) vs 36 (4.8%)	2.02 (1.28, 3.17)	0.002
IgG deposits in the mesangium + CLs vs in the mesangium only	17 (2.3%) vs 24 (3.2%)	2.51 (1.76, 3.01)	0.023

Values are presented as number (percentage).

Of the 41 patients with IgG co-deposition reaching the primary combined outcome, 17 (48.6%) had mesangium + CLs deposits (*p* < 0.01; Table 3). The chance of experiencing the primary combined outcome was 2.51-fold higher in patients with IgG deposits in the mesangium + CLs compared with those with IgG deposits in the mesangium only (95% CI: 1.76–3.01, *p* = 0.023; Table 3). Kaplan–Meier survival curves illustrated that the overall survival rate was significantly lower in patients with IgAN with IgG deposits in the mesangium + CLs than in those with IgG deposits in the mesangium only (log-rank test *χ*^2^ = 4.769, *p* = 0.029; Figure 3(B)).

In a multivariate Cox proportion model, IgG deposits in the mesangium + CLs remained independent predictors of renal outcome after adjusting for clinical variables such as age, sex, MAP, proteinuria, and eGFR (Model 2, Table 4). When the location of IgG deposits was included in a multivariate model comprising Oxford-MESTC and clinical variables, IgG deposits in the mesangium + CLs predicted worse primary combined outcomes independently of the clinical variables and Oxford-MESTC (Model 3, Table 4).

**Table 4.** Multivariate Cox regression models for the renal outcome of doubling of Scr concentration, 50% reduction in estimated glomerular filtration rate or renal outcome of end-stage renal disease in study subjects.

	Model 1^a^		Model 2^b^		Model 3^c^	
	HR (95% CI)	*p*-Value	HR (95% CI)	*p*-Value	HR (95% CI)	*p*-Value
Age (per 1 year)	1.01 (0.99, 1.03)	0.232	0.98 (0.96, 1.00)	0.132	0.98 (0.96, 1.01)	0.295
Women (vs men)	1.36 (0.89, 2.06)	0.148	1.55 (1.01, 2.39)	0.045	1.50 (0.85, 2.67)	0.066
Mean arterial pressure (per 1 mmHg)			1.02 (1.00, 1.03)	0.020	1.02 (1.01, 1.04)	0.024
Proteinuria (per 1 g)			1.06 (0.96, 1.26)	0.224	1.05 (0.92.1.20)	0.351
Estimated glomerular filtration rate (per 1 ml/min/1.73 m^2^)			0.97 (0.96, 0.98)	0.000	0.97 (0.96, 0.98)	0.000
M1 (vs M0)					NS	NS
E1 (E0)					1.05 (0.55, 2.00)	0.835
S1 (vs S0)					1.20 (0.64, 2.27)	0.451
T1 (vs T0)					1.06 (0.43, 2.56)	0.875
T2 (vs T0)					2.62 (1.87, 7.89)	0.025
C1-2 (vs C0)					1.18 (0.65, 2.16)	0.477
IgG deposits in Mesangium only (vs IgA without IgG deposition)	1.85 (1.13, 3.03)	0.020	1.30 (0.74, 2.28)	0.361	1.37 (0.63, 2.95)	0.287
IgG deposits in Mesangium and CLs (vs IgA without IgG deposition)	1.93 (1.11, 3.37)	0.013	2.03 (1.20, 3.34)	0.008	2.11 (1.06, 4.18)	0.005

HR: hazard ratio; CI: confidence interval; NS: not significant; M0: mesangial hypercellularity score of ≤0:5; M1: mesangial hypercellularity score of >0:5; E0: absence of endocapillary hypercellularity; E1: presence of endocapillary hypercellularity; S0: absence of segmental glomerulosclerosis; S1: presence of segmental glomerulosclerosis; T0: tubular atrophy/interstitial fibrosis ≤25% of cortical area; T1: tubular atrophy/interstitial fibrosis 26–50% of the cortical area; T2: tubular atrophy/interstitial fibrosis >50% of cortical area; C0: absence of crescents; C1-2: presence of crescents.

^a^Model 1: age, sex, and the location of glomerular IgG deposit; ^b^model 2: model 1+ baseline clinical variables (mean arterial pressure, proteinuria, estimated glomerular filtration rate); ^c^Model 3: model 2 + M, E, S, T. C.

## Discussion

The purpose of this study is two-fold: (i) to determine if the presence or absence of IgG co-deposition in patients with IgAN is indeed predictive of poor renal outcome in a large cohort and (ii) to determine if the location of glomerular IgG deposits exhibits a progressive course in IgAN.

In the present study, we conducted a retrospective analysis of the data of 742 primary patients with IgAN. Our study showed that 182 of 742 patients had positive instances of IgG staining (positive rate 24.5%), consistent with the rates of 15–80% reported in the literature [21]. Our findings indicate that the presence of glomerular IgG co-deposition in IgAN and the location of IgG deposits in the mesangium + CLs portend severe clinical and pathological lesions and worse primary combined outcomes. Importantly, the multivariate Cox regression model analysis confirmed that only the location of glomerular IgG deposits in the CLs was an independent risk factor for poor prognosis in IgAN.

To date, the underlying mechanisms of IgAN with glomerular IgG deposits have not been elucidated completely. Berger and Hinglais first described IgA nephropathy (IgAN) in 1968 [22]. Previous studies suggested that galactose-deficient IgA1 (Gd-IgA1) acts as a ‘trigger’ in the pathogenesis of IgAN [23,24]. Specifically, IgAN is associated with the production of Gd-IgA1 recognized by IgG and/or IgA1 autoantibodies, resulting in the formation of immune complexes that deposit in the glomerular mesangium and incite glomerular damage, leading to proteinuria and hematuria [25–27]. Interestingly, Rizk et al. [28] found that even IgAN kidney-biopsy specimens without IgG by routine immunofluorescence microscopy had Gd-IgA1–specific IgG autoantibodies, we, therefore, speculate that the presence of mesangial IgG deposition might be determined by the amount of IgG deposition or the intensity of Gd-IgA1-IgG immune complexes or the timing of renal biopsy. However, our findings could not confirm these results, and further research is needed to confirm these findings. Bellur et al. [29] reported that IgAN with IgG co-deposition was associated with endocapillary hypercellularity and a higher mesangial cellularity score. Gabriel et al. [30] demonstrated that patients with IgAN with glomerular IgG co-deposition had lower hemoglobin and serum triglyceride levels. Our findings suggested that patients with IgAN with IgG co-deposition had greater histologic activity in the form of E1 and poorer primary combined outcomes. All of these results indicated that glomerular IgG deposition in IgAN correlated with the severity of diseases.

Therefore, how does glomerular IgG deposition exacerbate IgAN? Previous studies reported a subclass restriction, with IgG mesangial isotypes being predominantly IgG1 and IgG3, in IgAN with glomerular IgG co-deposition [31]; IgG1 and IgG3 can recognize Gd-IgA1 to form immune complexes, and then the immune complexes activate complement via the classical pathway to potentiate tissue injury in IgAN [32,33]. In line with this theory, our study has demonstrated that patients with IgAN with IgG co-deposition had a more positive instance of C1q staining, the recognition molecule of the classical pathway of the complement system. Moreover, the severity of IgG co-deposition was mild (1+) in most of the patients in the IgA-IgG group, and renal survival was significantly decreased among patients with a higher intensity of IgG deposition. IgAN patients with IgG present by routine immunofluorescence microscopy typically have worse outcomes may simply mean that the amount of IgG is greater and there is more local activation of complement to potentiate tissue injury in IgAN. Meanwhile, we also found that patients with a higher intensity of glomerular C3 deposits or C1q deposits were also associated with a lower survival rate, which is in line with the prior findings [34–36].

A good deal of clinical research has been performed to assess the prognostic value of IgG co-deposition in IgAN. D’Amico critically analyzed the results of 23 valid studies on IgAN published over the past 20 years and indicated that glomerular IgG co-deposition was an independent risk factor for the prognosis of IgAN [37]. Wada et al. [14] found that patients with IgAN with IgG co-deposition presented serious clinical features, a lower complete remission rate, and resistance to treatment. Furthermore, glomerular IgG co-deposition was reportedly related to the development of hypertension during follow-up and was an independent determinant of progression in IgAN [38]. A recent follow-up study reported that glomerular IgG deposition correlated with greater histological activity and increased clinical severity and was independently associated with worse primary combined outcomes in patients with IgA nephropathy [39]. Our study confirms the finding of an association between glomerular IgG co-deposition and worse primary combined outcome.

More importantly, our findings also described that patients with IgAN with IgG deposits in the mesangium and CLs portended severe clinical and pathological lesions, indicated by lower eGFR, higher levels of MAP, proteinuria, serum creatinine, and serum total cholesterol at biopsy and higher proportions of a positive instance of IgM and C1q staining, endocapillary hypercellularity (E1), tubular atrophy/interstitial fibrosis (T1-2), crescents, small vessel hyalinosis and severe interstitial inflammation infiltration, as well as a higher possibility of reaching the primary combined outcome compared with patients with glomerular IgG deposits restricted to the mesangium. Additionally, multivariable analysis of the Cox model showed that a location of glomerular IgG deposits only in the CLs is an independent risk factor for poor prognosis in patients with IgAN. The clinical significance of the immune deposit location was also noted in previous studies. A Japanese study suggested that IgA-IgG subepithelial deposits may induce the rupture or increased permeability of the glomerular capillary walls, accompanied by thinning and splitting of the lamina densa and the formation of crescents of various sizes [40], which is consistent with our findings. Incidentally, Wada et al. [14] also found that patients with IgG deposits in the capillary wall presented a higher level of proteinuria and were far more prone to accept steroid pulse therapy combined with tonsillectomy. In an American multicenter cohort, Alvarado et al. [15] reported that glomerular immune deposits in the peripheral capillary walls were associated with a significantly increased risk of primary combined outcomes in IgAN. Additionally, we found that patients with IgAN with IgG deposits in the mesangium and CLs were associated with corticosteroid and immunosuppressant treatments, likely due to more severe clinical and pathological lesions.

Notably, IgG is a macromolecular protein that cannot pass through the walls of blood vessels. It may be reasonable to assume that immune deposits occur in the CLs under their molecular charge and size or their affinity for specific tissue moieties [41]. Interestingly, our study showed that patients with IgA with glomerular IgG co-deposition in the CLs had more positive instances of IgM staining. A previous study demonstrated that high levels of IgM in the circulation would activate suppressor T cells, leading to increased secretion of lymphokines such as vascular permeability factor, thus contributing to increased capillary permeability [42]. This may be one of the mechanisms responsible for glomerular IgG deposits in the CLs in IgAN and implies that patients with IgAN with glomerular IgG deposits in the CLs had more severe renal arteriole lesions. Moreover, our study confirms this speculation of an association between patients with IgAN with IgG deposits in the CLs and the severity of renal arteriole lesions such as renal arteriole wall thickening and hyalinosis.

This study has several limitations. First, the retrospective observational design of the study makes our results susceptible to selection bias, and a retrospective study depends on records, which may be incomplete. Furthermore, this was a single-center study, and because of the ethnic homogeneity, we cannot generalize these findings to other racial or ethnic groups. Third, previous research has suggested that IgG subtyping might be related to recurrence of infection, immunodeficiency, and other autoimmune diseases [33].

Most notably, we did not routinely perform IgG subtyping at biopsy to evaluate the significance of IgG subtyping in IgAN. Moreover, IgA nephropathy is a typical chronic disease, and our follow-up duration was too short to evaluate the patients’ prognoses. Last, we cannot exclude that residual confounding or unmeasured potential confounders may remain. Therefore, further long-term prospective studies are required to reveal the exact pathophysiologic mechanisms and clinical impacts of the presence of glomerular IgG deposits and the location of glomerular IgG deposits in patients with IgAN.

In conclusion, the present study demonstrated that the presence of glomerular IgG co-deposition and the location of glomerular IgG deposits in the CLs were both associated with severe clinical and pathological lesions at biopsy and worse renal outcome, but only glomerular IgG deposits in the CLs was an independent risk factor for poor prognosis in IgAN. Additionally, the intensity of glomerular IgG deposits or C3 deposits or C1q deposits was also related to prognosis in IgAN. We suggest that these findings should be taken into account for treatment decisions and the design of therapeutic trials.
